# Exploiting Gene-Expression Deconvolution to Probe the Genetics of the Immune System

**DOI:** 10.1371/journal.pcbi.1004856

**Published:** 2016-04-01

**Authors:** Yael Steuerman, Irit Gat-Viks

**Affiliations:** Department of Cell Research and Immunology, George S. Wise Faculty of Life Sciences, Tel Aviv University, Tel Aviv, Israel; The Jackson Laboratory, UNITED STATES

## Abstract

Sequence variation can affect the physiological state of the immune system. Major experimental efforts targeted at understanding the genetic control of the abundance of immune cell subpopulations. However, these studies are typically focused on a limited number of immune cell types, mainly due to the use of relatively low throughput cell-sorting technologies. Here we present an algorithm that can reveal the genetic basis of inter-individual variation in the abundance of immune cell types using only gene expression and genotyping measurements as input. Our algorithm predicts the abundance of immune cell subpopulations based on the RNA levels of informative marker genes within a complex tissue, and then provides the genetic control on these predicted immune traits as output. A key feature of the approach is the integration of predictions from various sets of marker genes and refinement of these sets to avoid spurious signals. Our evaluation of both synthetic and real biological data shows the significant benefits of the new approach. Our method, VoCAL, is implemented in the freely available R package ComICS.

## Author Summary

Quantitative trait locus (QTL) studies have identified a plethora of genetic variants that lead to inter-individual variation in the abundance of immune cell subpopulations, both in normal and disease states. Cell sorting is an effective method of monitoring immune cell type quantities; however, owing to the large number of possible immune cell subsets, it can be difficult to apply this method to each cell type over multiple individuals. Recent QTL studies dealt with this difficulty by focusing on an *a priori* selection of one or a few cell subsets. Here we introduce VoCAL, a deconvolution-based method that utilizes transcriptome data to infer the quantities of immune cell types, and then uses these quantitative traits to uncover the underlying DNA loci. Our results in synthetic data and lung cohorts show that the VoCAL method outperforms other alternatives in revealing the genetic basis of immune physiology.

## Introduction

The immune system consists of a remarkable collection of immune cell subpopulations with complex interconnections. To gain a better understanding of immune processes at the cellular level, such as cell proliferation, differentiation, activation and migration, researchers have systematically quantified the abundance of particular immune cell types in health and disease. This approach has provided insights into the role of immune cells during both homeostasis and disease progression; for example, recruitment and accumulation of macrophages in adipose tissue are associated with obesity [1]; the presence of eosinophils in the airway lumen and lung tissues is considered a defining feature of asthmatic disease [2]; recruitment of monocytes to arterial vessel walls is an early step in the development of atherosclerosis [3]; and an increase in CD4^+^CD28^null^ T cells is detectable in patients with complications of rheumatoid arthritis [4]. There is a strong need for workable methodological approaches that can identify the underlying molecular mechanisms determining the physiological state of the immune system. A major goal in this endeavor is to identify genetic variants that lead to inter-individual variation in the abundance of particular immune cell types.

In studying the genetic basis of immune physiology, both genotyping and immune-cell quantification must be performed and analyzed in concert. Direct measurement of the abundance of a large number of immune cell types remains a challenge because of the relatively low throughput of cell-sorting technologies. Such direct quantification is particularly laborious when a large number of individuals is studied, and as a result, most association studies are restricted to only a few immune-cell types [5–17], with few exceptions [18–20]. Thus, a simplified approach is required.

With the advent of immune deconvolution methods, it is now possible to infer the relative abundance of immune cell subpopulations without the need for experimental cell sorting. Specifically, deconvolution methods take as input expression profiles of isolated immune cell types (in short, a 'reference data'; *e*.*g*., [21–24]) and an expression profile from a complex tissue. The expression of each gene in a tissue is modeled as a linear combination of its expression in each cell type, where the weights stand for the unknown abundance of each immune cell type. This abundance can be resolved by solving a set of linear equations, one for each gene. Previous studies have shown that using only a subset of carefully selected genes (rather than the whole expression signature) typically reduces the signal-to-noise ratio and stabilizes the solution (*e*.*g*., [24,25]). For example, 360, 61, 240 and 547 genes were selected for immune cell type deconvolution in [24–27], respectively. The selected genes, which are used as observations during the deconvolution process, are referred to as 'markers'. Deconvolution techniques have been successfully applied to predict the composition of immune cell types, but have not yet been applied in the context of genetic studies.

We describe here a method for revealing the genetic basis of inter-individual variation in the abundance of immune cell types. Our method relies on a deconvolution algorithm that receives as input expression profiles from a complex tissue across a population of individuals, and uses this data to calculate relative cell type abundance values in each individual. The underlying genetic variants are then identified on the basis of the predicted cell type abundance levels, without the need for experimental cell quantification. In our framework, the predicted abundance of a particular cell type across a certain cohort of individuals is termed an 'immune trait'; the associated DNA variant is referred to as an 'immune quantitative trait locus' (iQTL); and the genetic association is termed an 'immune trait association'. Since we use predicted traits (rather than direct measurements), special care has to be taken to ensure the reliability of the identified immune trait associations. To that end, we resample several disjoint sets of marker genes and then repeat the pipeline using the different marker sets. An association is considered reliable if it attains high significance, on average, based on several different sets of marker genes. We also realized that part of the reason for false positive iQTLs is the presence of genetic control on the expression levels of marker genes (such genomic loci are typically termed 'expression QTLs' [eQTLs]). To overcome this difficulty, we filter out marker genes that are associated with potentially misleading eQTLs. Our rationale is to discriminate between true iQTLs and spurious ones: a false positive iQTL (due to an eQTL) can be eliminated by removing the relevant eQTL targets from the marker set; true iQTLs, in contrast, are generally robust to such alterations in the set of marker genes. We refer to this approach as the VoCAL (Variation in Cell Abundance Loci) algorithm.

We used synthetic data to assess the performance of the VoCAL algorithm in a controlled setting. Using these data, we start by demonstrating the increasing complexity of the iQTL-identification problem with increasing numbers of eQTLs in a tissue. We next show the utility of VoCAL over a large range of data parameters, and demonstrate the benefits of discarding potentially misleading eQTLs while combining evidence from multiple sets of markers. As a proof of principle, we applied VoCAL to genotyping and lung expression profiles from recombinant inbred BXD mouse strains, thereby demonstrating the ability of VoCAL to identify significant iQTLs while removing spurious associations.

## Results

### Motivation

**Fig 1:**
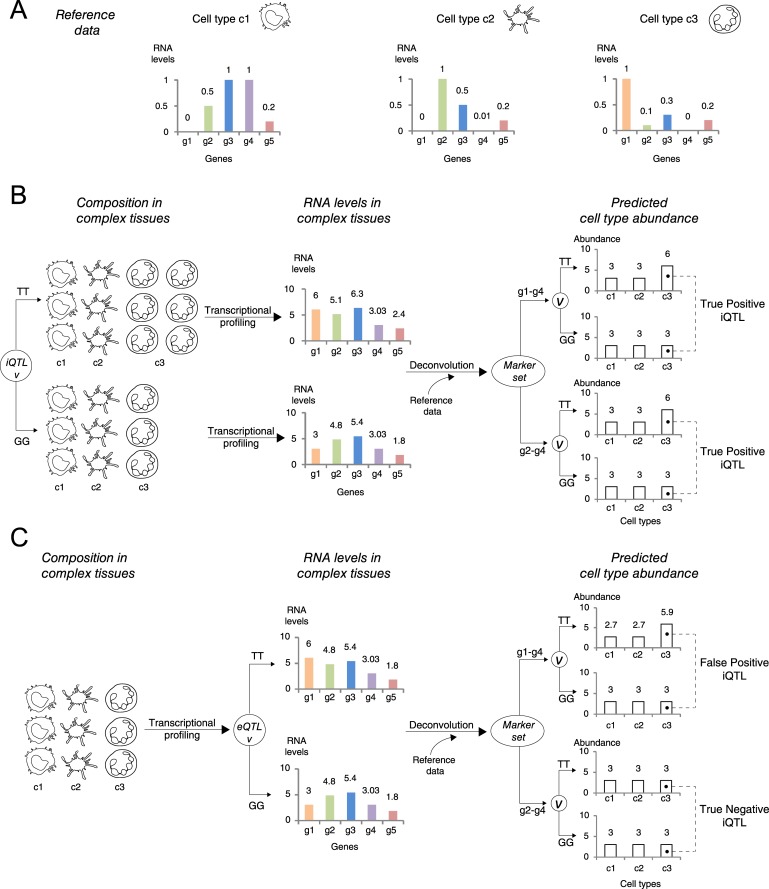
Revealing the genetic basis of inter-individual variation in the abundance of immune cell types in a complex tissue. (**A**) A simple reference data. Shown are three cell types (*c1-c3*, left to right); each cell type consists of five genes (*g1-g5*), and its transcriptional profiling is described as a bar plot. (**B**,**C**) Each scheme consists of a certain composition of cell types in a tissue (left) and the transcriptional profiling of the same tissue (middle). The right panel provides the output of a computational deconvolution process (that is, the inferred abundance of each immune cell type in the tissue) given transcriptional profiling (middle), a reference data (from **A**), and a certain marker set. Here we exemplify the *g1-g4* marker set (top right) and the *g2-g4* marker set (bottom right). (**B**) A conceptual scheme of an iQTL acting on the quantity of cell type *c3* (left). Transcriptional profiling is exploited by the deconvolution procedure to correctly reveal difference in the abundance of cell type *c3* between genotypes, which is subsequently identified as a true positive iQTL (right). The two marker sets *g1-g4* and *g2-g4* are shown to provide similar predictions. (**C**) A scheme of an eQTL *v* acting on the RNA level of gene *g1* (middle). Using the eQTL target *g1* as a marker results in distinct quantities of *c3* between the genotypes, which leads to a spurious *v-c3* association (using *g1-g4* as markers, top right). Notably, this erroneous association can be eliminated by removing the eQTL target *g1* from the marker set (using *g2-g4* as markers, bottom right). In **B** and **C**, all RNA levels and inferred cell type abundance values were calculated using a linear regression model (using Eq 1 in Methods).

In the following we consider how to find, in the absence of direct cell-sorting measurements, the genetic basis of immune traits. To address this we rely on a computational inference of cell type abundance levels from gene expression data. We begin with an illustrated example to explain the basic rationale of this approach and follow with the actual pipeline of the VoCAL methodology. Consider a simplified reference data consisting of transcriptional profiles from three immune cell types (*c1-c3*), assuming that each cell type contains only five genes (*g1-g5;* Fig 1A). In this reference data, each plot describes the RNA levels of each gene in each cell type. For example, the plots indicate the cell type-specific expression of gene *g1* in cell type *c3*.

The scenario in Fig 1B (left) considers a certain genomic locus *v* that has an effect on the abundance of cell type *c3* within a given tissue. This locus is therefore an iQTL. In accordance, Fig 1B (left) demonstrates the higher level of cell type *c3* in TT-carrying compared to GG-carrying individuals. The plots of total RNA levels in the tissue are shown in the middle panel of Fig 1B; these RNA levels reflect the composition of cell types in the tissue and the RNA levels within each cell type. As can be seen in the figure, if a TT-carrying individual has an increased abundance of cell type *c3*, then its level of the *c3*-specific gene *g1* is also elevated (Fig 1B, middle).

Mathematical deconvolution methods take as input a certain list of marker genes, and then use the total RNA levels of these markers in the complex tissue to calculate the abundance of each cell type. In our example, the inferred (deconvolved) cell type quantities are shown in the right panel of Fig 1B, for each genotype and for two potential sets of marker genes (marker sets *g1-g4* [top] and *g2-g4* [bottom]); this prediction relies on the total RNA levels in the tissue from Fig 1B (middle panel) and the reference data from Fig 1A. It can be clearly seen that each of the two marker sets can be utilized to correctly predict (i) a higher abundance of cell type *c3* in TT-carrying individuals, and (ii) a similar amount of cell types *c1* and *c2* in different genetic backgrounds (Fig 1B, right). Thus, by repeating the same deconvolution process in multiple individuals, it is possible to identify true immune trait associations (*e*.*g*, *v*-*c*3) and to reject false ones (*e*.*g*., *v-c1* and *v-c2*); furthermore, we expect that the identification of true associations will be generally robust to the selection of marker genes. This observation is key to the success of the VoCAL algorithm.

While studying the system, we discovered a potential pitfall of this approach—the existence of eQTLs acting on the intracellular RNA levels of genes. To gain some intuition about why this is the case, consider the presence of an eQTL acting in locus *v* (instead of an iQTL in this genomic position). For instance, Fig 1C shows an effect of eQTL acting on the expression of gene *g1*. We see that the TT- and GG-carrying individuals differ only in their RNA level of gene *g1* (Fig 1C, middle) but not in their composition of cell types (Fig 1C, left). Yet, when a marker set *g1-g4* is used, a deconvolution algorithm may output an erroneous increased abundance of cell type *c3* in TT-carrying individuals, which might be interpreted as an association between locus *v* and cell type *c3* (a false positive iQTL; Fig 1C, top right). We note that the spurious association stems from *g1* (a *c3*-specific marker and an eQTL target) and can be eliminated by excluding *g1* from the marker set (*e*.*g*., marker set *g2-g4*, Fig 1C, bottom right). Thus, the inclusion of eQTL targets in the set of marker genes may interfere with our algorithm and lead to spurious iQTLs at the same genomic positions. Following this rationale, construction of marker sets that do not include eQTL targets can, in principle, be used to avoid spurious predictions. The VoCAL algorithm relies on this idea, as discussed below.

### The VoCAL Algorithm

**Fig 2:**
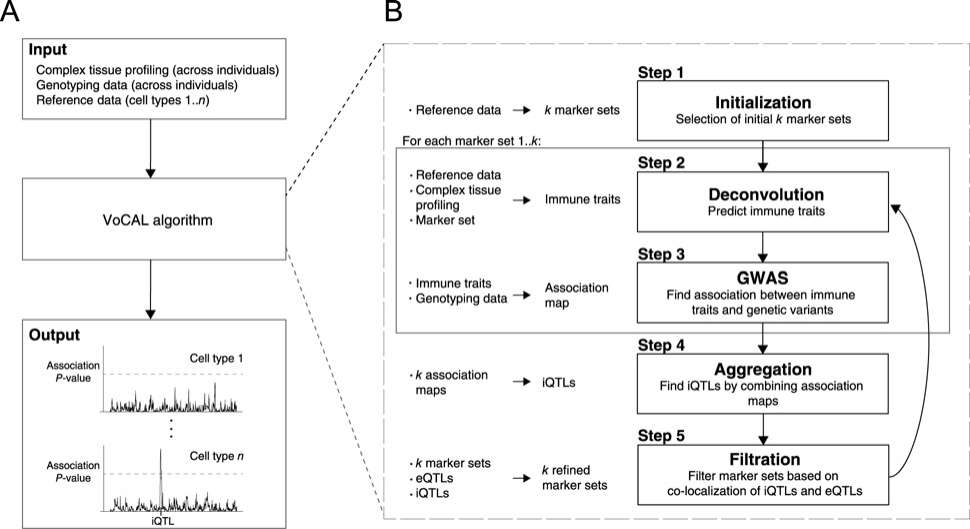
Flow chart of the VoCAL algorithm. (**A**). An overview of the analysis. VoCAL takes as input gene expression profiles in complex tissues from a population of genotyped individuals and a reference data containing expression profiles of isolated immune cell types (top box). VoCAL utilizes this input to identify significant 'immune trait associations'—the associations between immune cell type abundance levels and their underlying iQTLs. (**B**) The VoCAL pipeline. VoCAL proceeds in five steps: step 1—choosing the initial *k* sets of marker genes; step 2—predicting immune traits (namely, cell type quantities across individuals) based on the reference data and gene expression of the marker genes in a tissue; step 3—mapping the association between each immune trait and each genomic locus. VoCAL generates replicates of this association map by applying steps 2–3 repeatedly using each of the *k* (non-overlapping) marker sets from step 1; step 4—consolidating the collection of association maps into combined association *P*-values between each immune trait and each locus. Significantly associated loci are referred to as iQTLs; step 5—filtration of the *k* marker sets to exclude potentially confounding eQTL targets. If at least one marker is filtered out, VoCAL returns to step 2.

We devised the VoCAL method with the specific object of using deconvolution to identify significant associations between cell type abundance traits and polymorphic DNA loci. The input of the VoCAL algorithm is the gene expression profiles of a given complex tissue across a population of genetically distinct (genotyped) individuals, as well as a large 'reference data' of transcriptional profiles from isolated immune cell subsets (Fig 2A, top). The output is a collection of significant iQTLs (Fig 2A, bottom).

As we discussed, VoCAL relies on two observations. First, we expect noisy predictions to be weakly reproducible between marker sets, but true iQTLs to be consistently identified by multiple different marker sets. Based on this rationale, VoCAL combines iQTL predictions from multiple marker sets to produce a reliable model. Second, eQTL targets may lead to spurious iQTL associations. Naively, VoCAL could filter all eQTL targets in a pre-processing step. However, a potential caveat of this strategy is that the removal of many informative markers might reduce the ability to detect iQTLs. To address this, VoCAL leverages the observation that the expression of misleading markers likely associates with eQTLs located within the inferred iQTLs (*e*.*g*., locus *v* in Fig 1C). The problem is a challenging one, as the identification of iQTLs requires the selection of markers, and the selection of markers requires knowledge about the genomic positions of iQTLs. This necessitates the identification of both the iQTLs and the gene markers simultaneously. To address this, VoCAL applies an iterative approach. In each iteration, VoCAL uses the sets of selected markers to identify iQTLs and then uses the identified iQTLs to filter out confounding markers.

In particular, VoCAL consists of five steps (Fig 2B). In step 1—*initialization*—VoCAL constructs an initial collection of *k marker sets*. In this stage we do not yet have the inferred iQTLs. Thus, each set of markers is selected based on the ability of the genes to discriminate well between immune cell types in the reference data. This strategy has been proven useful in deconvolution of immune cell types [24–27]. Steps 2 and 3 are repeated *k* times, each time with a different set of markers. In step 2—*Deconvolution*—VoCAL relies on a mathematical deconvolution algorithm to predict cell type abundance levels. The input to this procedure is (i) the expression data of a complex tissue across individuals, (ii) the reference data, and (iii) a single set of marker genes. The output is a collection of *immune traits*, each consisting of inferred cell abundance values for a single cell type across the individual samples. In step 3—*genome-wide association testing (GWAS)*—VoCAL applies a statistical association test on each immune trait, producing association scores between each genomic locus and each immune trait. We term such a collection of association scores as an *association map*.

Altogether, steps 2 and 3 provide a collection of *k* association maps (a single map for each marker set). In step 4—*aggregation*—VoCAL combines the *k* association maps to produce a reliable model. In particular, for each given locus and each given immune trait, VoCAL calculates a single *association P-value* based on the relevant scores in the collection of *k* maps. Significantly associated loci are referred to as *iQTLs*. In step 5—*filtration*—VoCAL refines the *k* sets of marker genes by filtering out eQTL targets. Specifically, the filtration step tests whether any of the current marker genes is associated with an eQTL that coincides with an inferred iQTL. If such markers are found, VoCAL filters them out and returns to step 2.

In summary, the VoCAL procedure starts with an initial selection of *k* marker sets (step 1) and then iterates between two tasks: a reliable identification of significant iQTLs relatively to a given collection of *k* marker sets (steps 2, 3, and 4), and the filtration of marker sets relatively to the collection of significant iQTLs (step 5). The algorithm terminates when there are no more changes to the marker genes. A detailed description of the VoCAL algorithm appears in the Methods section. The associated R package ComICS is available at https://cran.r-project.org/web/packages/ComICS/index.html and csgi.tau.ac.il/VoCAL/.

### A Framework for Evaluating the Utility of the VoCAL Algorithm

To evaluate the performance of VoCAL, it was necessary to simulate iQTLs and eQTLs in synthetic complex tissues. To do this over a population of individuals, we used genotyping of the recombinant inbred BXD mouse strains (102 individuals) and a reference data containing expression profiles of isolated immune cell types (taken from the ImmGen project [23]). First we randomly selected one or a few cell types from this reference data and a polymorphic locus (an iQTL) for each of these cell types; groups of co-expressed genes sharing the same eQTL hotspots were selected in a similar manner. Next, assuming an initial equal abundance of cell types for each individual, we altered the fractions of the chosen cell types according to the DNA allele of the selected iQTL. The magnitude of the change in cell type fractions is termed the *iQTL effect size*. Lastly, we generated the final expression values of each tissue sample by (i) mixing the signatures from the reference data according to those fractions, and (ii) introducing the effect of the selected eQTLs on their target groups of genes (the magnitude of this effect is termed the *eQTL effect size*).

**Fig 3:**
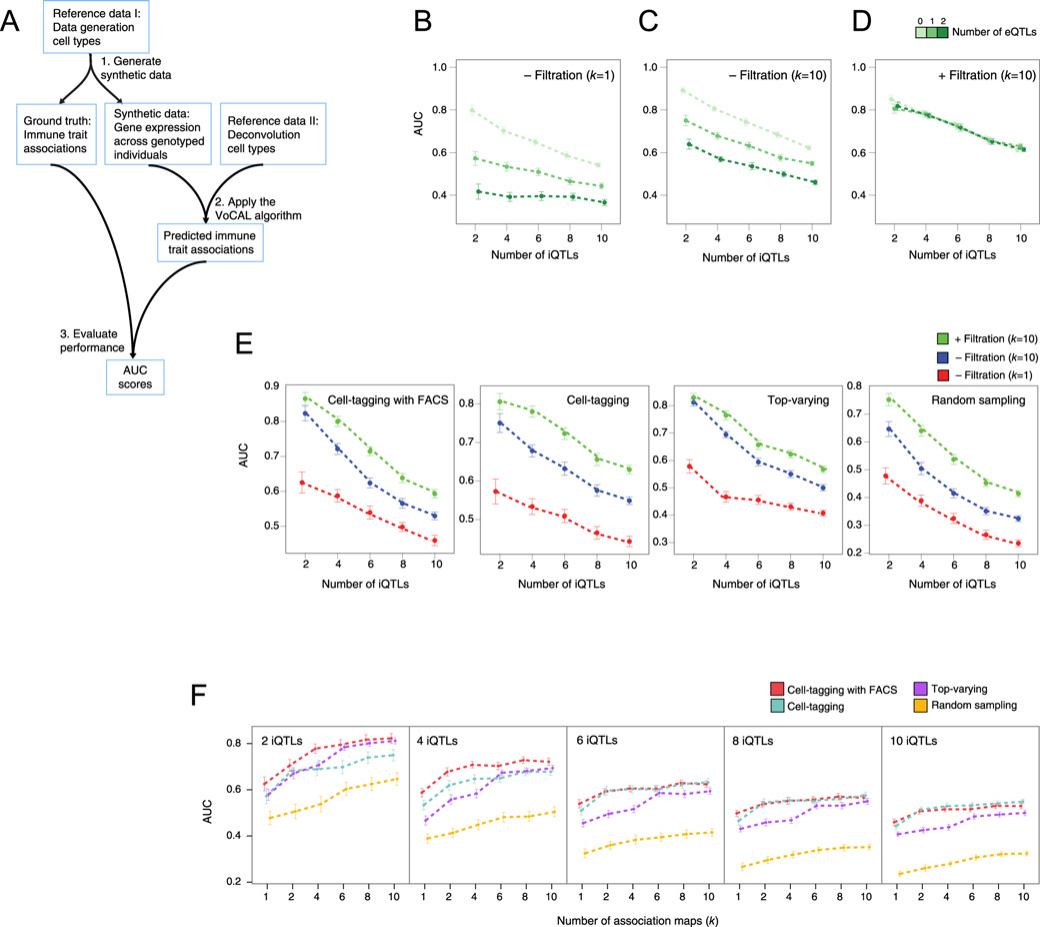
Performance of the VoCAL algorithm in synthetic data. (**A**) Overview of our performance evaluation pipeline. The simulation takes as input a collection of cell type signatures (the 'data generation cell types') and provides both synthetic data and a 'ground truth' solution. VoCAL is applied on this synthetic data using a reference data carrying 'deconvolution cell types' (thus we do not use exactly the same cell types for both data generation and deconvolution). Predicted and ground truth associations are then compared to assess the performance of the VoCAL algorithm (based on an AUC score). (**B-D**) Effect of the number of eQTLs and iQTLs. Shown are AUC scores (*y*-axis) for varying numbers of iQTLs (*x-*axis) and different numbers of eQTL hotspots (color-coded) using the cell-tagging method. We applied VoCAL (**B**) without filtration, *k* = 1, (**C**) without filtration, *k* = 10, and (**D**) with filtration, *k* = 10. (**E**) Performance evaluation using different initialization methods (subpanels). Reported are AUC scores (*y*-axis) for varying numbers of iQTLs (*x-*axis) and 1 eQTL hotspot. VoCAL was applied without filtration and *k* = 1 (red), without filtration and *k* = 10 (blue), and using filtration with *k* = 10 (green). (**F**) Improved performance using a larger number of association maps. The AUC measure (*y*-axis) was obtained using different numbers of association maps (*k*; *x*-axis) with four alternative initialization methods (color coded), different numbers of iQTLs (subpanels) and 1 eQTL hotspot. (**B-F**) As expected, the complexity of the problem grows with the larger amount of iQTLs or eQTLs. The plots clearly show that VoCAL performance improves when reproducibility is tested over a larger number of association maps (a larger *k*), and when the filtration step is applied. In all cases, iQTL- and eQTL-effect size = 0.05.

To account for the common scenario in which the cell types that are used during the deconvolution process are not exactly the same as the cell types in the complex biological tissue, we used two disjoint sets of cell types: one set is used for synthetic data generation (the 'data-generation cell types'), while the VoCAL algorithm—particularly the deconvolution process—was applied based on another set of cell types (the 'deconvolution cell types'; Fig 3A). Each cell type in one set is closely related to a cell type in the other set (for example, the same cell type isolated from different tissues; S1 Table), allowing us to use the ground truth immune-trait associations to evaluate the predictions of the VoCAL algorithm. Although the simulation may not perfectly mirror a real tissue, it can still provide a model for a tissue that is (i) affected by iQTLs and by eQTL hotspots leading to variation in specific cell types and genes, and (ii) characterized by cell types that are similar but not identical to the cell types given as input to the VoCAL algorithm (see Methods and S1 Fig).

Here we examine and demonstrate four different initialization methods (used in step 1 of the VoCAL algorithm): (i) choosing sets of gene markers carrying the highest variability in expression between cell types (*top varying)*; (ii) choosing representative marker genes that can discriminate well between cell types (*cell tagging;* 24); (iii) using the cell-tagging strategy but adding a predefined set of cell surface markers that were used in the cell-isolation process (*cell tagging with FACS;* 36); and (iv) using an unbiased selection of gene markers (*random sampling*). We note that the three former methods (but not random sampling) are based on the cell type signatures in the reference data.

The ability to identify the correct iQTLs was evaluated as the area under the receiver operating curve (*AUC score*). Notably, the results are robust to variation in the parameters of the VoCAL algorithm (S2 Fig). For example, different significance cutoff of the identified iQTLs (varying between 0.05 and 10^−12^) had a little effect on the eventual AUC scores. Motivated by these results, we analyzed the effect of different data parameters and the benefits of the VoCAL algorithm, as discussed below.

### Complexity of the Problem Increases with the Number of Causal Genetic Loci

We first investigated how the complexity of the problem is affected by the presence of eQTLs and iQTLs in a tissue. To assess this, we generated synthetic datasets with varying numbers of iQTLS and eQTLs, each of which acts through a fixed size of its genetic effect. Overall, we tested a total of 15 such combinations of different numbers of QTLs. First, we applied VoCAL without the 'filtration step' (using steps 1–4 only), allowing us to trace the effect of eQTL targets within the marker sets. As expected, predictions in datasets with smaller numbers of iQTLs were more accurate (Fig 3B). Furthermore, consistent with our expectation (Fig 1C), the ability to identify iQTLs depended not only on the number of iQTLs, but also on the number of eQTLs: the AUC scores were lower in datasets with higher numbers of eQTLs. For example, AUCs were significantly higher for datasets with no eQTL hotspots than for those with 2 eQTL hotspots for the same number of iQTLs (average AUC = 0.7 vs. 0.39; *P* < 2∙2^−16^ (*t*-test), in the presence of 4 iQTLs, effect sizes = 0.05, cell-tagging and *k* = 1; Fig 3B). These results were quantitatively similar when using a larger number of association maps (*k* = 10; Fig 3C) and for different initialization methods and data parameters (S3 Fig, left and middle panels). We conclude that the iQTL-identification problem becomes more complex with increasing amounts of different genetic effects; without applying the filtration step, the presence of eQTLs results in relatively low performance values.

### VoCAL Is Robust to the Presence of Confounding eQTLs in a Tissue

Next we were interested in the effect of applying the filtration step (step 5, Fig 2B). We found that the iterative filtration of marker sets improved the prediction of iQTLs. In particular, without filtration of eQTL targets, the presence of eQTLs in a tissue resulted in a drastic reduction in AUC scores (Fig 3B and 3C); in contrast, in the presence of the filtration procedure, there was little or no reduction in AUC scores when more eQTLs were added (Fig 3D). The same was true when different initialization methods and data types were used (*e*.*g*., Figs 3E and S3). Notably, marker filtration brought no improvement when the complexity was increased by multiple iQTLs (Fig 3D and 3E); this is consistent with the primary goal of the filtration procedure, which is to tackle the problem of confounding eQTLs (rather than the problem of interactions among multiple iQTLs).

To gain additional insights into the filtration step, we analyzed 2-dimensional plots of AUC scores for the same synthetic datasets with (*x*-axis) and without (*y*-axis) this step (S4A Fig). In the case of 2 eQTL hotspots, all datasets appear above the diagonal line, indicating that the filtration step resulted in improved performance (*e*.*g*., using the top-varying initialization method, *P* < 2∙10^−34^ (*t*-test); S4A Fig, left panel). In contrast, the AUC scores remained nearly unchanged when eQTLs were not introduced into the simulation (*e*.*g*., using cell-tagging with FACS and 10 iQTLs, *P* > 0.15 (*t*-test); S4A Fig, right panel). The patterns were similar when we used false positive rate (FPR) and true positive rate (TPR) metrics instead of the AUC (*e*.*g*., S4B and S4C Fig). Taken together, these results indicated that the filtration procedure successfully reduces the amount of spurious associations derived from the effects of eQTLs in a tissue.

### Reproducibility across Multiple Association Maps Contributes to the Performance of the VoCAL Algorithm

We next investigated the added value of generating *k* association maps rather than a single map. To that end, we compared the performance of VoCAL with 10 association maps to its performance with a single map. We found that the power to detect iQTLs increased drastically when using 10 association maps (Figs 3B and 3C, S3 and S5A). For example, using 1 eQTL and 6 iQTLs, the usage of 10 association maps is significantly better than using one selected map (*P* <2∙10^−16^, paired *t*-test; assuming effect size of 0.05, the cell-tagging method, without filtration). In fact, the AUC scores were quantitatively correlated with the number of association maps (Fig 3F). These results were qualitatively similar when using different initialization methods and different numbers of iQTLs (Figs 3F and S5B–S5D).

We further tested the possibility of pooling the *k* marker sets into a single large set. As a proof of principle, we focus on two alternative strategies. In the first strategy, VoCAL was applied using *k* association maps, where each map relies on a marker set consisting of Ψ markers. Alternatively, VoCAL was applied with a single marker set that was generated by pooling the *k* disjoint marker sets of the former method. Since we use the cell-tagging initialization method, the resulting pooled set is the same as direct selection of Ψ∙*k* cell-tagging markers. This way, both strategies were initialized with exactly the same list of markers. We find better performance with multiple marker sets as compared to a single pooled set (S6 Fig). For example, when we use *k* = 6 and 2 iQTLs, *P* < 5∙10^−11^ (*t*-test) for multiple sets over the pooled set. Taken together, our results demonstrate the benefit of testing reproducibility in association signals when relying on multiple non-overlapping marker sets.

### Performance Improvement with a Reference-Based Initialization of Marker Sets

We also compared the reference-based initialization methods—the top-varying and two tagging-based methods—with random sampling of marker sets. The reference-based selection of marker genes showed a striking improvement in performance over the random sampling of markers, especially when the number of iQTLs was large (*e*.*g*., S7 Fig). For example, when we used 8 iQTLs and 1 eQTL hotspot, *P* < 6∙10^−91^ (*t*-test) for cell-tagging over the random sampling approach. The results for different parameter settings were similar (*e*.*g*., S3 Fig). Thus, the current study clearly supports a rationalized initialization of marker sets. Notably, since one method of reference-based initialization did not seem to consistently outperform the others, we could not find a convincing reason to prefer one method over another.

### Identification of iQTLs in the Lung Tissue

**Fig 4:**
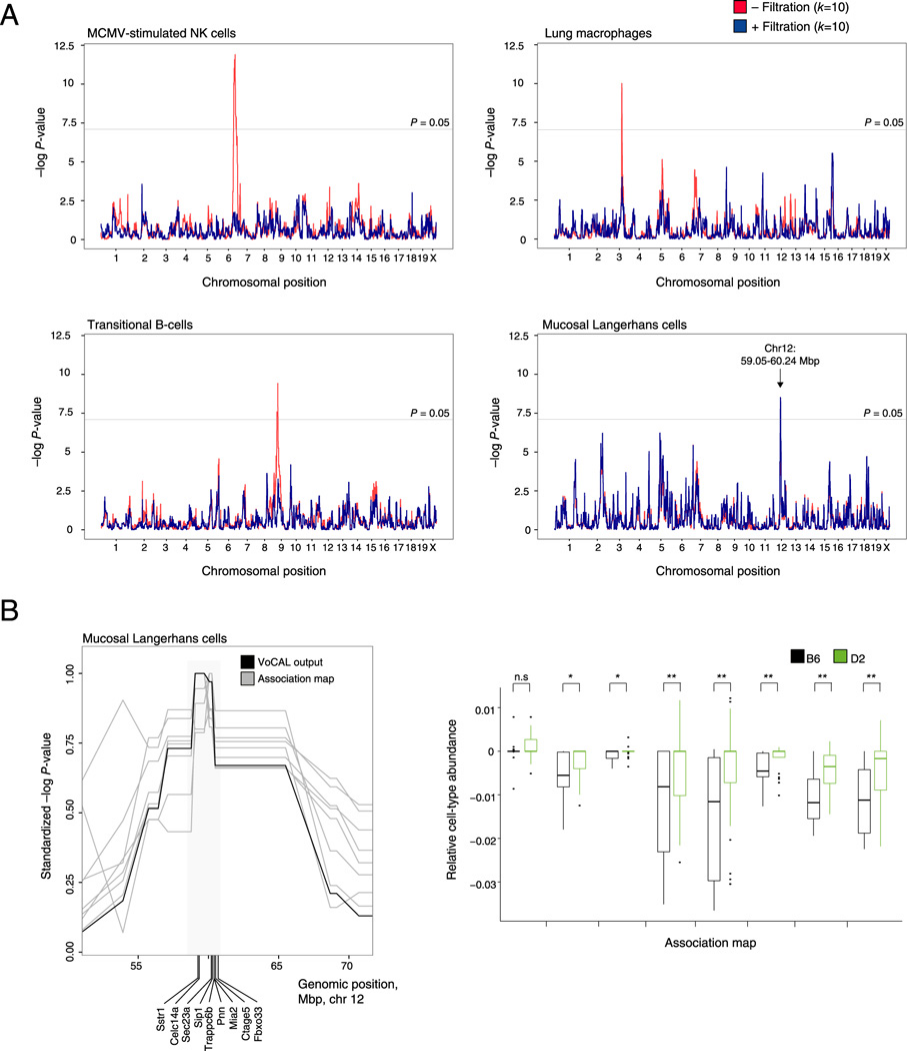
Analysis of lung transcriptomes reveals genetic control on mucosal Langerhans cells, demonstrating the benefits of the VoCAL algorithm. (**A**) Associations in the presence or absence of the filtration step. Shown are −log *P*-values of association generated by the VoCAL algorithm with (blue) or without (red) the filtration step (*y-*axis) across genomic positions (*x*-axis). Exemplified are natural killer (NK) cells (top left), lung macrophages (top right), B cells (bottom left) and mucosal Langerhans cells (bottom right). The results suggest a promising association of mucosal Langerhans cells (permutation FDR < 0.025; detailed in panel **B**), and highlights the ability of marker filtration to eliminate spurious associations in the remaining cell types. (**B**) Langerhans cells demonstrate the advantages of using multiple association maps. Presented are 8 association maps that were constructed by the VoCAL algorithm for mucosal Langerhans cells. Left panel:–log *P-*values of association tests (*y*-axis) of the 8 association maps (gray) and of the final VoCAL output (black) across genomic positions (*x*-axis). Scores were normalized by the maximal score of each association map. The plot demonstrates agreement between the different association maps. Right panel: Predicted abundance of mucosal Langerhans cells (*y*-axis), which were utilized to generate the 8 association maps (*x*-axis) in individuals carrying the B6 (black) or D2 (green) allele in the associated iQTL (the rs3705833 locus; *P < 0.01; **P < 0.001). In all cases, D2-carrying individuals exhibited a higher predicted abundance of Langerhans cells than did B6-carrying individuals.

We applied the VoCAL algorithm to identify iQTLs in the lung gene expression dataset of Alberts et al. [28], which was measured across a collection of (genotyped) naive BXD mouse strains (a cross of C57BL/6J [B6] and DBA/2J [D2] strains). The analysis was conducted using the 'cell-tagging with FACS' initialization method on the basis of the ImmGen reference data [23], which carries 207 immune cell types. VoCAL converged after three iterations, with removal of 13 and 3 markers in the first two iterations, respectively, and no additional filtration in the third. In the absence of marker filtration, 7 significant iQTLs were apparent, associated with the abundance of murine cytomegalovirus (MCMV)-stimulated natural killer (NK) cells, lung macrophages, mucosal Langerhans cells, non-classical MHC class II^int^ monocytes, effector T cells, transitional type 2 B cells, and B1a cells (permutation FDR < 0.05; see Fig 4A and full details in S2 and S3 Tables). However, only the Langerhans cells exhibited significant association when we applied VoCAL with the iterative filtration of marker genes (Fig 4A). On the assumption that our study with synthetic data was realistic, the six remaining associations probably indicate false positives, since they appeared only in the presence of a few eQTLs that could have stemmed from any of the cell populations in the tissue.

The subpopulation of MCMV-stimulated NK cells demonstrates VoCAL's ability to address the eQTL-confounding problem. In the absence of marker filtration step, these cells were found to be significantly associated with a 25.6-Mb region on chromosome 6, with a peak between 129.56–133.8 Mbp (Fig 4A, top left). The RNA levels of three marker genes—*Klrc3*, *Klrk1* and *Klra8*—were associated with an eQTL residing in the same iQTL region. In accordance, the three markers were removed during the filtration step and the association completely vanished (Fig 4A, top left). Consistent with our predictions, all three markers have a known role as NK-specific receptors, with a specific role of *Klra8* in MCMV infection [29–31]. Brown et al. [30] reported that (i) splenic NK cells are abundant in both the B6 and D2 strains (the parental strains of BXD lines); and (ii) in NK cells, the *Klra8* gene is expressed in B6 but not D2 mice. Furthermore, Lee *et al*. [31] showed that, using spleen and liver tissues, the *Klra8* gene could be amplified from the B6 strain but not from the D2 strain. Thus, our predictions in NK cells agree well with previous studies. Additional experiments are required to test the NK hypothesis in the lung tissue.

The mucosal Langerhans cells provide a clear example of a predicted iQTL (Fig 4B). In lung tissue, mucosal Langerhans cells act as the first line of defense against invading pathogens. Using VoCAL, the Langerhans cells were significantly associated with a 1.2-Mb region iQTL on chromosome 12 (from 59.05 to 60.24 Mbp) with permutation-based FDR < 0.025. The predicted iQTL interval consisted of 9 genes (Fig 4B, left), none of these genes had any *cis*-association. Notably, 2 of these 9 genes located at the peak of this interval—somatostatin receptor 1 (*Sstr1*) and C-type lectin domain receptor (*Clec14a*)—have documented roles in Langerhans cells (*e*.*g*., [32–34]). The association with Langerhans cells also demonstrates the advantages of aggregating *k* association maps, as the results consisted primarily of consistent association pattern (8 out of 10 independently derived maps; Fig 4B, left). These maps were in agreement with the overall prediction of the VoCAL algorithm (Fig 4B, left, black line). Furthermore, in all of these cases strains carrying the D2 allele showed higher predicted quantities of Langerhans cells than strains carrying the B6 allele (Fig 4B, right and S4 Table; based on the rs3705833 locus located at chromosome no. 12 at 59.05 Mbp). This highlights a major advantage of our approach: true iQTLs are expected to be revealed on the basis of distinct subsets of markers. The independent support of the iQTL interval from different marker sets and the lack of eQTLs in this region are in agreement with our hypothesis of an iQTL acting on Langerhans cells in chromosome no. 12.

## Discussion

In this work we developed a novel method, which we call VoCAL, to reveal the genetic basis of variation in immune cell traits based on gene expression data. Whereas existing methods for genetic mapping require direct measurement of immune traits across a large population of individuals, VoCAL avoids cell quantification by inferring these immune traits indirectly. To address this, VoCAL utilizes a mathematical deconvolution technique, which relies on a set of marker genes, to calculate the abundance of a variety of immune traits; it then applies genome-wide association methods to uncover the causal loci for these traits (iQTLs). By consolidating hypotheses from different marker sets we avoid errors from noisy predictions (Fig 2B). This technique relies on the observation that true signals are generally robust to the choice of a marker set, as demonstrated in Fig 1B. Our analysis indeed demonstrates the improved performance of this approach (Figs 3F, S5 and S6) and the consistency between predictions derived from distinct marker sets in the murine lung-tissue dataset (Fig 4B).

Suspecting that the existence of eQTL targets may lead to spurious iQTL associations (as demonstrated in Fig 1C), the VoCAL pipeline refines the selected sets of markers by filtering out potentially confounding eQTL targets (Fig 2B). Our analysis in synthetic data confirms the increased complexity of the problem with increasing number of eQTLs (Figs 3B–3D and S3) and the improved performance when using the filtration step (Figs 3D and 3E and S4). Analysis of a biological dataset from the lung complex tissue further underscores the utility of the filtration step: of the seven putative associations found, only one still holds after filtration of eQTL targets (Fig 4A), suggesting that the remaining associations are purely due to confounding eQTLs. For example, we discovered that the association of a subpopulation of NK cells was eliminated when the *Klra8* gene was removed from the set of marker genes. This prediction is in agreement with previous *in vitro* measurements [30,31]. Taken together, our results emphasize VoCAL's ability to eliminate spurious associations that do not reflect an actual change in quantity of an entire cell subpopulation, but rather an inter-individual variation in expression of particular genes.

Our findings point the way to several avenues of research. First, additional methods capable of dealing with biological tissues of high genetic complexity will have to be developed; for example, joint analysis of several iQTLs and eQTLs may enhance predictive power and make it possible to distinguish immune cell-cell and gene-cell interactions. Second, it will be important to extend VoCAL to human data. For example, we should use a human reference data (such as [35]); account for different confounding effects such as population structure and gender; and extend the association tests (Eq 2) to handle heterozygous populations. Third, taking into account the correlations between the different marker genes might enhance our predictive power. Fourth, manipulation of the reference data (as in [36]) would allow us to explore genetic loci that lead to a shift of an immune cell to its inflammatory state. Fifth, it would be important to incorporate environmental effects, thereby highlighting the role of non-heritable factors in physiological immune responses.

Finally, the ability to predict iQTLs provides plausible hypotheses for future experimental investigations. For example, this study suggests an iQTL acting on immune Langerhans cells located at the lung mucosa. The association holds when using multiple different marker sets and after applying the marker filtration step (Fig 4B). Langerhans cells play a key role in innate defense against pathogens, suggesting a framework for understanding the genetic and immune cell interactions underlying susceptibility to respiratory infections. Additional investigations are needed to explore the functionality of changes in the abundance of these cells in the lung tissue. Overall, the methodology is general and can be applied with other deconvolution tools (*e*.*g*., [27]), and for other applications in the mammalian immune system.

## Methods

### The VoCAL Algorithm

VoCAL takes as input the following information:

1. *A reference data X* in the form of an *m* × *n* matrix whose *x*_*j*,*c*_ entry is the expression value of gene *j* ∈ {1,…,*m*} in cell type *c* ∈ {1,…,*n*}.
2. RNA profiling of a complex tissue *Y* in the form of an *m* × *l* matrix whose *y*_*j*,*s*_ entry is the expression of gene *j* ∈ {1,…,*m*} in individual *s* ∈ {1,…,*l*}.
3. Genotyping data. In a genome of *q* loci we use the variable *v* ∈ {1,…,*q*} to index each position. A *genetic matrix D* is an *l* × *q* matrix whose *d*_*s*,*v*_ entry is the genotype of individual *s* in genomic position *v*. *d*^(*v*)^ is a column vector of genotyping in locus *v* across the *l* individuals.

The VoCAL pipeline involves five steps: initialization, deconvolution, GWAS, aggregation and filtration. In the following we first describe the details of the different steps and then provide the overall VoCAL algorithm. A brief summary of the VoCAL framework is provided in S8 Fig.

#### Initialization of marker genes (step 1)

A selected *marker set u* is defined as *F*^(*u*)^ ⊆ *F*, where *F* is the set of all genes. The initialization step generates *k* different marker sets ${\left\{F^{\left(u\right)}\right\}}_{u=1}^{k}\subseteq F$. Our underlying assumption is that the intersection between different marker sets *F*^(*i*)^ and *F*^(*j*)^ is *F*^(*i*)^ ∩ *F*^(*j*)^ = *ϕ*. To address this, we start with an initial selection of markers *F*^(1)^ and then choose the marker sets one after the other, where the *i*-th marker set (*i* > 1) is selected from among the genes that were left after the previous selection *F*^(*i*)^ ∈ *F* \ {*F*^(1)^ ∪⋯∪ *F*^(*i*−2)^ ∪ *F*^(*i*−1)^}. Each of the next steps (steps 2 and 3) runs *k* times, once for each of the *k* selected marker sets.

Diverse marker selection methods can be used to initialize the marker sets. Here we tested four different methods: (i) The *top-varying* method selects *k* sets of markers, each of which carries the highest variability of expression between cell types in the reference data. (ii) The *cell-tagging* method selects *k* marker sets, where each set can discriminate well between cell types in the reference data, as previously described by Abbas *et al*. [24]. The generation of each marker set proceeds as follows: for each gene, each cell type is ranked (from high to low) according to its gene-expression levels, and a standard *t*-test is computed between the top cell type and the second-top and third-top cell types; the selected markers are those genes carrying the best *t*-test *P*-values for each cell type. (iii) The *cell-tagging with FACS* methodology first utilizes a predefined FACS-based subset of immune cell surface markers that were used in the experimental sorting of immune cells in the reference data [25]. It then adds *k-*1 cell-tagging marker sets. Finally (iv), the *random sampling* method selects *k* marker sets, each of which consists of an arbitrary selection of genes. In this study, the number of markers in each set, denoted Ψ, is the same across all initialization methods.

#### Deconvolution (step 2)

Given a set of markers *F*^(*u*)^, we now consider how to estimate the abundance level of each immune cell type (namely, predicting immune traits). Step 2 relies on the column vector *y*^(*s*)^ = (*y*_1,*s*_,…,*y*_*m*,*s*_)' of the normalized *expression data* of all *m* genes in individual *s*. Since we rely only on a selected marker set *F*^(*u*)^, it is possible to eliminate all genes in the input datasets *X* and *y*^(*s*)^ that are in *F* \ *F*^(*u*)^; the resulting data is denoted *X*^(*u*)^ and *y*^(*s*,*u*)^, respectively. Based on this data, a deconvolution technique can be applied as previously proposed [37]: for a certain marker set *F*^(*u*)^ and individual *s*, the fitting of cell type abundances levels is formalized as a regression problem of the form:

$$y^{\left(s,u\right)}=X^{\left(u\right)}\cdot \theta^{\left(s,u\right)}\;.$$ (1)

In particular, $\theta^{\left(s,u\right)}=(\theta_{1}^{\;(s,u)},\ldots ,\theta_{n}^{\;(s,u)})$ where $\theta_{c}^{\left(s,u\right)}$ represents the abundance of cell type *c* in individual *s* (assuming a given marker set *u*). We define an *immune trait* of cell type *c* as the predicted abundance of this cell type across all individuals (a column vector $\theta_{c}^{\left(u\right)}$).

It should be noted that in some cases the data consists of a large number of cell types in addition to the large number of genes. The marker selection steps (steps 1 and 5) reduce the number of genes, but the large number of cell types remains a challenge. For example, in our real data analysis, the ImmGen reference data [23] consists of *n* = 207 cell types, whereas the number of markers can be smaller (*e*.*g*., as in the case of the FACS-based marker set, where Ψ = 61). The challenge is twofold: firstly, the large number of cell types leads to over-parameterization, and secondly, we assume that it is biologically unlikely to expect changes in the abundance of a large fraction of the cell types under study. In recent studies, we tackled this problem by regularizing the linear regression from Eq 1 to choose a subset of cell types providing the best fit to the expression of markers (the DCQ algorithm; [25,38]). In these previous studies, the regularization was done via the elastic net regression [39] using the glmnet implementation [40] with regularization parameters lambda.min.ratio = 0.2 and α = 0.05. Notably, additional analysis showed that the accuracy of deconvolution is robust to the particular selection of parameters [25]. Motivated by this observation, here we applied the glmnet elastic net with the same parameters (see details in S2A Fig). Taken together, VoCAL utilizes two complementary approaches to ensure robustness of the deconvolution output: it applies deconvolution on a carefully selected set of genes (steps 1 and 5) while penalizing for quantity changes in a large number of immune cell types (step 2).

#### Genome-wide association study (GWAS) of immune traits (step 3)

VoCAL calculates an association score for each genomic locus and each immune trait. The input is a matrix of immune traits $\theta^{\left(u\right)}=(\theta_{1}^{\left(u\right)},\ldots ,\theta_{n}^{\left(u\right)})$ (the output of step 2 assuming a certain marker set *F*^(*u*)^ from step 1) as well as a genotyping matrix *D*. Diverse association tests can be applied [41]. In this study we used a simple linear ANOVA model for the immune trait of cell type *c* being tested for its association with a homozygous genomic locus *v*, where *β*_*v*,*c*_ is a fixed effect and *ε* ~ *N*(0,*σ*^2^) is a random effect:

$$\theta_{c}^{\left(u\right)}=d^{\left(v\right)}\cdot \beta_{v,c}+\varepsilon .$$ (2)

We note that Eq 2 could be reformulated to account for covariates (such as age and gender) and heterozygous population. The resulting *P*-value for a given locus *v* and cell type *c* is denoted *p*_*u*_(*c*,*v*). The entire set of *P*-values *P*_*u*_ = {*p*_*u*_(*c*,*v*)|*c* = 1,…,*n*, *v* = 1,…,*q*} is referred to as an *association map*. Notably, steps 2 and 3 are applied *k* time, once for each marker set *F*^(*u*)^ from step 1, thus providing a collection of *k* association maps.

#### Aggregation of association signals (step 4)

To identify reliable associations, we search for significant *P*-values that are highly recurrent in the collection of *k* association maps. To that end, VoCAL utilizes the Fisher's combined probability test to attain the output *association P-values*, *a*_*c*,*v*_, for each cell type *c* and locus *v*:

$$a_{c,v}=\text{Pr}\;ob(\chi_{2k}^{2}\geq -2\sum_{u=1}^{k}\text{ln}\;p_{u}(c,v)),$$ (3)

where $\chi_{2k}^{2}$ denotes the *χ*^2^ distribution with *2k* degrees of freedom. Based on this definition, let *W*_*i*_ be a set *of significant immune trait associations*, meaning a collection of pairs (cell type *c* and locus *v*) where −log *a*_*c*,*v*_ is bigger than a certain significance cutoff *T*_*i*_. Formally, *W*_*i*_ = {(*c*,*v*)|−log *a*_*c*,*v*_ ≥ *T*_*i*_}.

#### Filtration of the marker sets (step 5)

The filtration step requires knowledge about eQTLs. To address this, the association between the expression of each gene *g*_*j*_ and each genomic locus *v* is calculated in a pre-processing step using the ANOVA linear model *y*_*j*_ = *d*^(*v*)^ ⋅ *β*_*v*,*j*_ + *ε*. Here, *y*_*j*_ = (*y*_*j*,1_,…,*y*_*j*,*l*_)' is a column vector of the expression of gene *g*_*j*_ in all individuals; *β*_*v*,*j*_ is a fixed effect of a locus *v*; and *ε* is a random effect. The ANOVA's corresponding *P*-values are denoted *p*(*g*_*j*_, *v*). The set of *significant expression trait associations*, *W*_*e*_, is defined accordingly by *W*_*e*_ = {(*g*_*j*_, *v*)|−log *p*(*g*_*j*_, *v*) ≥ *T*_*e*_}, with *T*_*e*_ indicating a certain significance threshold.

The filtration step is done as follows: VoCAL first constructs a set *F'* of potentially confounding genes that should be removed from the *k* markers sets. Specifically, *F'* includes those genes that are significantly associated with an eQTL that is also associated with a certain immune trait:

$$F'=\{g_{j}|\exists (c,v)\in W_{i}\;s.t.\;(g_{j},v)\in W_{e}\;\}.$$ (4)

Next, each marker set *F*^(*u*)^ is filtered by setting *F*^(*u*)^ = *F*^(*u*)^ \ *F*'.

#### Summary of the VoCAL algorithm

The algorithm starts with an initial selection of a collection of *k* marker sets ${\left\{F^{\left(u\right)}\right\}}_{u=1}^{k}\subseteq F$ (step 1). It then repeatedly applies the two iterative tasks: (i) identification of significant immune traits associations (the *W*_*i*_ set; steps 2, 3 and 4), and (ii) filtration of the marker sets by setting *F*^(*u*)^ = *F*^(*u*)^ \ *F*' (step 5). In each iteration we filter out markers according to the learned immune trait associations (*W*_*i*_) and then re-optimize the associations relatively to the filtered marker sets. The algorithm terminates when no additional filtration is needed (see outline of the VoCAL algorithm in Figs 2 and S8).

### Benchmarking and Performance Analysis

#### A general framework for estimating the performance of the VoCAL algorithm

To allow for a situation common in real biological data, where the reference cell types may differ slightly from the cell types in the actual tissue, and to avoid overfitting, we used different sets of cell types during (i) synthetic data generation and (ii) deconvolution process (step 2), as part of the VoCAL algorithm. The former set is referred to as the set of *data generation cell types*, and the latter as the set of *deconvolution cell types* (see Fig 3A). The two cell type sets were generated in two steps: (i) the different cell types in the ImmGen reference transcriptome data [23] were divided into groups according to the specific cell-surface markers used in their isolation process (as specified in [42]); and (ii) from each group we randomly chose 2 representative cell types such that one is placed in the set of data generation cell types and the other in the set of deconvolution cell types. The resulting total number of cell types in each set is *n = 31* (for details see S1 Table).

Each set of *n* cell types is used to construct a reference data. The *data generation reference data R* = {*r*_*j*,*c*_} is an *m*-by-*n* matrix whose *r*_*j*,*c*_ entry is the expression of gene *g*_*j*_ in a data generation cell type *c*. The *deconvolution reference data X* = {*x*_*j*,*c*_} is also an *m* × *n* matrix, where the *x*_*j*,*c*_ entry is the expression of *g*_*j*_ in a deconvolution cell type *c*. *X* is taken as input by the VoCAL algorithm and *R* is used to generate the collection of synthetic datasets. Given *X*, *D*, and synthetic data *Y* as input, VoCAL was applied with Ψ = 2∙*n =* 62, *T*_*i*_ = 10, *T*_*e*_ = 40 and 1 filtration step (*m* = 21,717). In the following we describe the generation of synthetic gene expression data *Y*.

#### Synthetic data generation

The simulation demonstrates different scenarios of changes in the iQTL-induced abundance of cell types, as well as changes in the expression of groups of genes associated with one or a few eQTL hotspots. The simulation is based on genotyping data of 3796 SNPs in the 102 recombinant inbred BXD mouse strains generated by crossing of the B6 and D2 parental strains [downloaded from GeneNetworks (http://www.genenetwork.org)]. Here, out of the 102 strains, each synthetic dataset consists of 60 BXD strains selected randomly (without replacements). Accordingly, each simulation takes as input a certain *l* × *q* matrix *D*, where the genotyping is given across *l* = 60 BXD strains and *q* = 3796 genomic loci. The simulation further takes as input the data generation reference *R*, as well as the required number of iQTLs (*n*_*i*_) and the number of eQTL hotspots (*n*_*e*_) and their respective effect sizes *γ*_*i*_ and *γ*_*e*_.

Transcription profiles were generated as follows: we first randomly chose *n*_*i*_ genomic loci that would act as iQTLs. A total of *n*_*e*_ eQTL hotspots were selected in the same manner. For each selected locus we arbitrarily chose the particular direction (activation or repression) of its action in the D2 allele. In addition, for each iQTL we randomly selected a target cell type *c* (from the set of data generation cell types). A group of 10 (co-expressed) target genes was selected arbitrarily for each eQTL hotspots (as described below). Let *I*_*a*_ and *I*_*r*_ be the 2 sets of cell types that are activated or repressed, respectively. Equivalently, the 2 sets of genes that are activated or repressed by eQTL hotspots are respectively denoted *E*_*a*_ and *E*_*r*_. To generate the synthetic dataset *Y* = {*y*_j,*s*_ | *j* = 1,…,*m*, *s* = 1,…,*l*}, each entry is calculated as:

$$y_{j,s}=\sum_{c=1,\ldots ,n}\theta_{c}^{\left(s\right)}\cdot r_{j,c}+\varphi_{j}{}^{\left(s\right)}+\varepsilon \;,$$ (5)

where *r*_*j*,*c*_ is the expression of gene *g*_*j*_ in cell type *c* and *ε* ~ *N*(0, *σ*^2^) is random noise. $\theta_{c}^{\left(s\right)}$ is the abundance of cell type *c* in strain *s*, possibly affected by an eQTL, and $\varphi_{j}^{\left(s\right)}$ represents potential alterations in the expression of a gene *g*_*j*_ in strain *s* due to an underlying eQTL. We assume that a locus (either an iQTL or an eQTL) has an effect only in those strains carrying the allele of the parental strain D2. Therefore, the formulations of $\theta_{c}^{\left(s\right)}$ and $\varphi_{j}^{\left(s\right)}$ are given by

$$\theta_{c}^{\left(s\right)}=\tilde{\theta_{c}^{\left(s\right)}/}\sum_{c=1,\ldots ,n}\tilde{\theta_{c}^{\left(s\right)}}$$ (6)

$$\tilde{\theta_{c}^{\left(s\right)}}=\left\{ \begin{array}{ll} \frac{1}{\left|C\right|} & \;c\notin \{I_{a},I_{r}\}\;\text{or}\;d_{s,v(c)}=B6\\ \frac{1}{\left|C\right|}-(\frac{1}{\left|C\right|}\cdot \gamma_{i}) & \;c\in I_{r}\;\text{and}\;d_{s,v(c)}=D2\\ \frac{1}{\left|C\right|}+(\frac{1}{\left|C\right|}\cdot \gamma_{i}) & \;c\in I_{a}\;\text{and}\;d_{s,v(c)}=D2 \end{array}\right.$$ (7)

$$\varphi_{j}^{\left(s\right)}=\left\{ \begin{array}{l} 0\;g_{\text{j}}\notin \{E_{a},E_{r}\}\;\text{or}\;d_{s,v(g_{\text{j}})}=B6\\ -\gamma_{e}\;g_{\text{j}}\in E_{r}\;\text{and}\;d_{s,v(g_{\text{j}})}=D2\\ \;\gamma_{e}\;g_{\text{j}}\in E_{a}\;\text{and}\;d_{s,v(g_{\text{j}})}=D2 \end{array}\;,\right.$$ (8)

where *v(c)* is the selected iQTL underlying the abundance of cell type *c* and *v*(*g*_*j*_) is the selected eQTL underlying the expression of a gene *g*_*j*_. Overall, we use equation nos. 5−8 to simulate 100 gene expression datasets (100 *Y* matrices) for each combination of the following parameters: number of iQTLs (*n*_*i*_ = 2, 4, 6, 8, 10), iQTL-effect size (*γ*_*i*_ = 0.05, 0.5), number of eQTLs (*n*_*e*_ = 0, 1, 2), and eQTL-effect size (*γ*_*e*_ = 0.05, 0.5). In all cases, *σ*^2^ = 0.0001.

We next consider the selection of groups of co-expressed target genes. In a preprocessing step, we combined all markers sets into a single list of genes. We then performed hierarchical clustering based on the expression of those genes in the reference data *R*, and split the clustering tree into sub-trees using a Pearson correlation cutoff > 0.7. A *group of target genes* is defined as a sub-tree consisting of more than 10 genes. During the generation of synthetic datasets, when we need to select a group of target genes for each given eQTL hotspot, we first randomly select the group of target genes and then randomly choose 10 genes from this group.

### Real Data

We analyzed the gene-expression profiling of whole lung tissue samples obtained from 47 BXD recombinant inbred mouse strains (E-MTAB-848 [28]). These strains were originally generated by crossing the B6 and D2 inbred strains, which are also included in this dataset. Using log-transformed data, we normalized each strain by subtracting the expression profile of the B6 strain. We used the 207 cell type profiles that are part of the ImmGen reference data (log-transformed; [23]). Genotyping data were reported and released in the GeneNetwork website (http://www.genenetwork.org). The genome annotations were based on UCSC Mouse Genome Browser NCBI37/mm9 assembly (RefSeq mm9). We applied VoCAL using the 'cell-tagging with FACS' initialization method with *k* = 10, *T*_*i*_ = 5 and *T*_*e*_ = 10.

We used 100 permutations of the labeling of strains in the lung expression data to assess the empirical FDR, defined as the ratio of the average number of associations found in the permuted data to the number of associations in the real lung data (denoted *'permutation FDR'*). We note that VoCAL utilizes permutations tests in addition to the resampling of markers (as detailed in step 1). The two procedures were designed to address two distinct challenges: whereas the selection of markers addresses the problem of noisy associations due to confounding eQTLs, the permutations aims to account for the multiple testing problem.

## Supporting Information

S1 Fig A comparison between our own performance evaluation scheme and a standard one. We compare two strategies: (i) Our own performance evaluation scheme, in which different cell types are used for data generation and for the deconvolution process (illustrated in Fig 3A); we refer to this scheme as the 'partition-based' approach. (ii) A naïve strategy, in which the same cell types are used for the purpose of data generation and the deconvolution step. (**A**) Comparative performance analysis. Means and standard deviations of AUC values (*y*-axis) in synthetic datasets with varying numbers of iQTLs (*x*-axis) are presented across 4 initialization methods (subpanels). Results are shown with the partition-based (red) and naïve (black) performance evaluation methods. (**B**) AUC of the VoCAL algorithm (*y*-axis) using the partition (left) and naïve (right) performance evaluation methods. In each plot, AUC values based on the random sampling approach are shown against any other initialization method (*x*-axis). In plots (**A**, **B**), results are shown over five synthetic datasets with *k* = 1 and iQTL effect size = 0.05, without any eQTL effect. Plots in (**A**) clearly show that AUC scores based on the naïve evaluation scheme are higher than those of the partition scheme (paired *t*-test *P* < 10^−112^), suggesting over-optimism of the naïve scheme. Plot (**B**) shows that the over-optimistic AUC scores of the naïve approach are particularly pronounced in the case of random sampling: in several model parameters, our partition-based simulation results in low (poor) AUC scores for the random sampling approach, whereas the naïve simulation over-optimizes the random sampling approach.
(EPS)

S2 Fig Parameter selection for the VoCAL algorithm. (**A**) Shown are the AUC scores (color coded) across different regularization parameters α (*x*-axis) and lambda.min.ratio (*y* axis) of the glmnet package [40]. The results were calculated without filtration, using the cell-tagging with FACS (left) and top-varying (right) initialization methods. (**B**) Shown are the AUC scores (*y*-axis) when using various *T*_*i*_ cutoffs (*x*-axis) across different initialization methods (color coded). *T*_*i*_ is defined as the–log immune trait association *P*-value cutoff (Methods). In both a and b we used synthetic data with 4 iQTLs, 1 eQTL hotspot, and iQTL- and eQTL- effect size = 0.05. The plots indicate the robustness of VoCAL over a large range of parameters. The selected parameters, which were used throughout this study, are highlighted in red.
(EPS)

S3 Fig Effect of the number of eQTLs and iQTLs. Shown are AUC scores (*y*-axis) for varying numbers of iQTLs (*x-*axis) and different numbers of eQTL hotspots (color-coded) using different initialization methods: (**A**) cell-tagging with FACS, (**B**) top-varying, (**C**) random sampling, and (**D**) cell-tagging. We applied VoCAL without filtration and *k* = 1 (left), without filtration and *k* = 10 (middle), as well as with filtration and *k* = 10 (right). In plots (**A-C**) iQTL- and eQTL-effect size = 0.05; in plot (**D**) iQTL- and eQTL-effect size = 0.5.
(EPS)

S4 Fig Marker filtration contributes to performance of the VoCAL algorithm. Summary of VoCAL's performance in the absence (*x*-axis) and presence (*y*-axis) of a marker filtration step. Scatter plots depict (**A**) the AUC scores, (**B**) the false-positive rate (FPR), and (**C**) the true-positive rate (TPR; *y*-axis), for synthetic datasets carrying 2 eQTL hotspots (left) or without eQTLs (right), over different initialization methods (subpanels). Results are shown (**A**) over varying numbers of iQTLs (turquoise scale), and (**B-C**) across varying association *P*-value thresholds using red or purple scale for 4 or 8 iQTLs, respectively. In all cases, iQTL- and eQTL-effect size = 0.05 and *k* = 10. The plots show that in the presence of eQTLs, AUC, FPR and TPR values are substantially improved when the filtration is applied; in contrast, in the absence of eQTLs, the AUC and TPR scores are relatively consistent and the FPR is only slightly improved.
(EPS)

S5 Fig Improved performance using a larger number of association maps. (**A**) Comparison of AUC scores obtained from VoCAL (*y*-axis) when using *k* = 10 association maps (blue) or *k* = 1 maps (red) across 4 and 8 iQTLs (*x*-axis). Results for each initialization method are shown in a different subpanel. In all cases, combining association maps from a larger number of repeats achieves significantly higher accuracy (*P* < 10^−35^). (**B-D**) Plots are shown as in Fig 3F, but using (**B**) no eQTL, iQTL-effect size = 0.05, no filtration; (**C**) 1 eQTL hotspot, effect size = 0.05, with filtration; and (**D**) 1 eQTL hotspot, iQTL- and eQTL-effect size = 0.5, no filtration.
(EPS)

S6 Fig Better performance with multiple smaller marker sets than a single larger one. Comparison of AUC scores obtained from VoCAL (*y*-axis) when using a single marker set with Ψ∙*k* markers (dashed), or *k* marker sets, each of which contains Ψ markers (solid) across various *k* values (*x*-axis). Results for varying numbers of iQTLs are shown in a different subpanel. All cases utilized cell-tagging, 1 eQTL hotspot, Ψ = 62, iQTL- and eQTL-effect size = 0.05, without filtration.
(EPS)

S7 Fig The accuracy of VoCAL is improved by the use of a rationalized (reference-based) initialization of marker sets. AUC (*y*-axis) obtained with different numbers of iQTLs (*x*-axis) without any eQTL effect. Depicted are four alternative initialization methods (color coded). In all cases, lower performance is achieved with random selection of initial marker sets than with the three reference-based methods.
(EPS)

S8 Fig Outline of the VoCAL algorithm. The outline details the identification of significant iQTLs given a certain collection of marker sets (**A**) and the overall VoCAL algorithm (**B**).
(EPS)

S1 Table Data generation and deconvolution cell type used in our simulation. Each row shows 2 immune cell subpopulations that were isolated using the same cell-surface markers (column 1; as annotated in [42]). One of these subpopulations is included in the data-generation set (columns 2, 3) and the other is part of the deconvolution set (columns 4, 5).
(XLSX)

S2 Table Predictions of the VoCAL algorithm in the lung tissue. Shown are all cell types that attained significant associations before or after application of the filtration step. For each cell type (column 1), the table records the cell-surface markers (column 2), the cell type description (column 3), the predicted iQTL interval (permutation FDR < 0.05; column 4), and the −log *P*-value of association at the peak of this predicted interval in the absence (column 5) or presence (column 6) of the filtration procedure.
(XLSX)

S3 Table A summary of the identified eQTL targets in the lung tissue. For each iteration of the VoCAL algorithm (column 1), the table provides the number of eQTL targets (columns 2–3) and the number of eQTL targets associated with iQTL regions (columns 4–5). For each category, the table records the total number of targets (columns 2, 4) and the number of targets that are also within the sets of marker genes (columns 3, 5; in iteration 1, these are the initial sets; in iterations 2 and 3 the sets were already filtered). Thus, column 5 records the number of markers that were actually filtered by the VoCAL algorithm.
(XLSX)

S4 Table Predicted association maps of mucosal Langerhans cells. For each association map (columns 1, 2), the table records the mean inferred relative abundance of Langerhans cells in individuals carrying the B6 allele (column 3) or the D2 allele (column 4), and the −log *P*-value of association (column 5) in locus rs3705833.
(XLSX)

## References

[1] McNelis J, Olefsky J. Macrophages, Immunity, and Metabolic Disease. Immunity. 2014 pp. 36–48. 10.1016/j.immuni.2014.05.010 25035952

[2] Uhm TG, Kim BS, Chung Y. Eosinophil development, regulation of eosinophil-specific genes, and role of eosinophils in the pathogenesis of asthma. Allergy, Asthma and Immunology Research. 2012 pp. 68–79. 10.4168/aair.2012.4.2.68 PMC3283796 22379601

[3] Mestas J, Ley K. Monocyte-Endothelial Cell Interactions in the Development of Atherosclerosis. Trends in Cardiovascular Medicine. 2008 pp. 228–232. 10.1016/j.tcm.2008.11.004 19185814 PMC2650852

[4] Yen JH, Moore BE, Nakajima T, Scholl D, Schaid DJ, Weyand CM, et al Major histocompatibility complex class I-recognizing receptors are disease risk genes in rheumatoid arthritis. J Exp Med. 2001;193: 1159–1167. 10.1084/jem.193.10.1159 11369787 PMC2193323

[5] Ferreira MAR, Hottenga J-J, Warrington NM, Medland SE, Willemsen G, Lawrence RW, et al Sequence variants in three loci influence monocyte counts and erythrocyte volume. Am J Hum Genet. 2009;85: 745–9. 10.1016/j.ajhg.2009.10.005 19853236 PMC2775836

[6] Nalls MA, Couper DJ, Tanaka T, van Rooij FJA, Chen MH, Smith A V., et al Multiple loci are associated with white blood cell phenotypes. PLoS Genet. 2011;7 10.1371/journal.pgen.1002113 PMC3128114 21738480

[7] Kelada SNP, Aylor DL, Peck BCE, Ryan JF, Tavarez U, Buus RJ, et al Genetic analysis of hematological parameters in incipient lines of the collaborative cross. G3 (Bethesda). 2012;2: 157–65. 10.1534/g3.111.001776 22384394 PMC3284323

[8] Cvejic A. From genome-wide association study hits to new insights into experimental hematology. Exp Hematol. 2014;42: 630–6. 10.1016/j.exphem.2014.04.005 24746874

[9] Soranzo N, Spector TD, Mangino M, Kühnel B, Rendon A, Teumer A, et al A genome-wide meta-analysis identifies 22 loci associated with eight hematological parameters in the HaemGen consortium. Nat Genet. 2009;41: 1182–90. 10.1038/ng.467 19820697 PMC3108459

[10] Phillippi J, Xie Y, Miller DR, Bell T a, Zhang Z, Lenarcic a B, et al Using the emerging Collaborative Cross to probe the immune system. Genes Immun. 2014;15: 38–46. 10.1038/gene.2013.59 24195963 PMC4004367

[11] Li J, Glessner JT, Zhang H, Hou C, Wei Z, Bradfield JP, et al GWAS of blood cell traits identifies novel associated loci and epistatic interactions in Caucasian and African-American children. Hum Mol Genet. 2013;22: 1457–64. 10.1093/hmg/dds534 23263863 PMC3657475

[12] Crosslin DR, McDavid A, Weston N, Zheng X, Hart E, de Andrade M, et al Genetic variation associated with circulating monocyte count in the eMERGE Network. Hum Mol Genet. 2013;22: 2119–27. 10.1093/hmg/ddt010 23314186 PMC3633369

[13] Ferreira MAR, Mangino M, Brumme CJ, Zhao ZZ, Medland SE, Wright MJ, et al Quantitative trait loci for CD4:CD8 lymphocyte ratio are associated with risk of type 1 diabetes and HIV-1 immune control. Am J Hum Genet. 2010;86: 88–92. 10.1016/j.ajhg.2009.12.008 20045101 PMC2801744

[14] Sankaran VG, Orkin SH. Genome-wide association studies of hematologic phenotypes: a window into human hematopoiesis. Curr Opin Genet Dev. 2013;23: 339–44. 10.1016/j.gde.2013.02.006 23477921 PMC4711360

[15] Rutledge H, Aylor DL, Carpenter DE, Peck BC, Chines P, Ostrowski LE, et al Genetic Regulation of Zfp30, CXCL1, and Neutrophilic Inflammation in Mouse Lung. Genetics. 2014; 10.1534/genetics.114.168138 PMC4196624 25114278

[16] Cusanovich DA, Billstrand C, Zhou X, Chavarria C, De Leon S, Michelini K, et al The combination of a genome-wide association study of lymphocyte count and analysis of gene expression data reveals novel asthma candidate genes. Hum Mol Genet. 2012;21: 2111–23. 10.1093/hmg/dds021 22286170 PMC3315207

[17] Raj T, Rothamel K, Mostafavi S, Ye C, Lee MN, Replogle JM, et al Polarization of the effects of autoimmune and neurodegenerative risk alleles in leukocytes. Science. 2014;344: 519–23. 10.1126/science.1249547 24786080 PMC4910825

[18] Orrù V, Steri M, Sole G, Sidore C, Virdis F, Dei M, et al Genetic variants regulating immune cell levels in health and disease. Cell. 2013;155 10.1016/j.cell.2013.08.041 PMC5541764 24074872

[19] Roederer M, Quaye L, Spector TD, Nestle FO, Roederer M, Quaye L, et al The Genetic Architecture of the Human Immune System: A Bioresource for Autoimmunity and Disease Pathogenesis. Cell. 2015;161: 1–17. 10.1016/j.cell.2015.02.046 PMC4393780 25772697

[20] Brodin P, Jojic V, Gao T, Bhattacharya S, Angel CJL, Furman D, et al Variation in the Human Immune System Is Largely Driven by Non-Heritable Influences. Cell. 2015;160: 37–47. 10.1016/j.cell.2014.12.020 25594173 PMC4302727

[21] Novershtern N, Subramanian A, Lawton LN, Mak RH, Haining WN, McConkey ME, et al Densely interconnected transcriptional circuits control cell states in human hematopoiesis. Cell. 2011;144: 296–309. 10.1016/j.cell.2011.01.004 21241896 PMC3049864

[22] Abbas a R, Baldwin D, Ma Y, Ouyang W, Gurney a, Martin F, et al Immune response in silico (IRIS): immune-specific genes identified from a compendium of microarray expression data. Genes Immun. 2005;6: 319–331. 10.1038/sj.gene.6364173 15789058

[23] Heng TSP, Painter MW. The Immunological Genome Project: networks of gene expression in immune cells. Nat Immunol. 2008;9: 1091–1094. 10.1038/ni1008-1091 18800157

[24] Abbas AR, Wolslegel K, Seshasayee D, Modrusan Z, Clark HF. Deconvolution of Blood Microarray Data Identifies Cellular Activation Patterns in Systemic Lupus Erythematosus. PLoS One. 2009;4: 16 10.1371/journal.pone.0006098 PMC2699551 19568420

[25] Altboum Z, Steuerman Y, David E, Barnett-Itzhaki Z, Valadarsky L, Keren-Shaul H, et al Digital cell quantification identifies global immune cell dynamics during influenza infection. Mol Syst Biol. 2014;10: 720 10.1002/msb.134947 24586061 PMC4023392

[26] Gong T, Hartmann N, Kohane IS, Brinkmann V, Staedtler F, Letzkus M, et al Optimal deconvolution of transcriptional profiling data using quadratic programming with application to complex clinical blood samples. PLoS One. 2011;6: e27156 10.1371/journal.pone.0027156 22110609 PMC3217948

[27] Newman AM, Liu CL, Green MR, Gentles AJ, Feng W, Xu Y, et al Robust enumeration of cell subsets from tissue expression profiles. Nat Methods. 2015;12: 453–457. 10.1038/nmeth.3337 25822800 PMC4739640

[28] Alberts R, Lu L, Williams RW, Schughart K. Genome-wide analysis of the mouse lung transcriptome reveals novel molecular gene interaction networks and cell-specific expression signatures. Respir Res. 2011;12: 61 10.1186/1465-9921-12-61 21535883 PMC3105947

[29] Daniels K a, Devora G, Lai WC, O’Donnell CL, Bennett M, Welsh RM. Murine cytomegalovirus is regulated by a discrete subset of natural killer cells reactive with monoclonal antibody to Ly49H. J Exp Med. 2001;194: 29–44. 10.1084/jem.194.1.29 11435470 PMC2193438

[30] Brown MG, Dokun a O, Heusel JW, Smith HR, Beckman DL, Blattenberger E a, et al Vital involvement of a natural killer cell activation receptor in resistance to viral infection. Science. 2001;292: 934–937. 10.1126/science.1060042 11340207

[31] Lee SH, Girard S, Macina D, Busà M, Zafer a, Belouchi a, et al Susceptibility to mouse cytomegalovirus is associated with deletion of an activating natural killer cell receptor of the C-type lectin superfamily. Nat Genet. 2001;28: 42–5. 10.1038/88247 11326273

[32] Harman AN, Bye CR, Nasr N, Sandgren KJ, Kim M, Mercier SK, et al Identification of lineage relationships and novel markers of blood and skin human dendritic cells. J Immunol. 2013;190: 66–79. 10.4049/jimmunol.1200779 23183897

[33] Hagforsen E, Michaëlsson G, Stridsberg M. Somatostatin receptors are strongly expresssed in palmoplantar sweat glands and ducts: Studies of normal and palmoplantar pustulosis skin. Clin Exp Dermatol. 2011;36: 521–527. 10.1111/j.1365-2230.2010.03993.x 21564193

[34] Hagströmer L, Emtestam L, Stridsberg M, Talme T. Expression pattern of somatostatin receptor subtypes 1–5 in human skin: An immunohistochemical study of healthy subjects and patients with psoriasis or atopic dermatitis. Exp Dermatol. 2006;15: 950–957. 10.1111/j.1600-0625.2006.00487.x 17083361

[35] Novershtern N, Subramanian A, Lawton LN, Mak RH, Haining WN, McConkey ME, et al Densely interconnected transcriptional circuits control cell states in human hematopoiesis. Cell. 2011;144: 296–309. 10.1016/j.cell.2011.01.004 21241896 PMC3049864

[36] Kidd BA, Wroblewska A, Boland MR, Agudo J, Merad M, Tatonetti NP, et al Mapping the effects of drugs on the immune system. Nat Biotechnol. 2015;34: 47–54. 10.1038/nbt.3367 26619012 PMC4706827

[37] Shen-Orr SS, Gaujoux R. Computational deconvolution: extracting cell type-specific information from heterogeneous samples. Curr Opin Immunol. 2013;25: 571–8. 10.1016/j.coi.2013.09.015 24148234 PMC3874291

[38] Frishberg A, Steuerman Y, Gat-Viks I. CoD: Inferring immune-cell quantities related to disease states. Bioinformatics. 2015;31: 3961–3969. 10.1093/bioinformatics/btv498 26315914

[39] Zou H, Hastie T. Regularization and variable selection via the elastic net. J R Stat Soc Ser B Stat Methodol. 2005;67: 301–320. 10.1111/j.1467-9868.2005.00503.x

[40] Friedman J, Hastie T, Tibshirani R. Regularization Paths for Generalized Linear Models via Coordinate Descent. J Stat Softw. 2010;33: 1–22. 20808728 PMC2929880

[41] Falconer DS, Mackay TFC. Introduction to Quantitative Genetics (4th Edition). 1996.

[42] Jojic V, Shay T, Sylvia K, Zuk O, Sun X, Kang J, et al Identification of transcriptional regulators in the mouse immune system. Nat Immunol. 2013;14: 633–643. 10.1038/ni.2587 23624555 PMC3690947

